# Essential Oil Composition and Enantioselective Profile of *Agastache urticifolia* (Lamiaceae) and *Monardella odoratissima* (Lamiaceae) from Utah

**DOI:** 10.3390/molecules28052249

**Published:** 2023-02-28

**Authors:** Tyler M. Wilson, Audra Davis, Reilly E. Sonstrom, Justin L. Neill, Emma A. Ziebarth, Ariel Poulson, Richard E. Carlson

**Affiliations:** 1 D. Gary Young Research Institute, Lehi, UT 84043, USA; 2 BrightSpec, Inc., Charlottesville, VA 22903, USA

**Keywords:** *Agastache urticifolia*, enantioselective profile, essential oil composition, gas chromatography, Lamiaceae, molecular rotational resonance (MRR), *Monardella odoratissima*

## Abstract

Two species within the Lamiaceae (mint) family, *Agastache urticifolia* and *Monardella odoratissima*, are aromatic plants that are native to the Intermountain Region (USA). Essential oil produced through steam distillation was examined to establish the essential oil yield and both the achiral and chiral aromatic profiles of both plant species. The resulting essential oils were analyzed by GC/MS, GC/FID, and MRR (molecular rotational resonance). For *A. urticifolia* and *M. odoratissima*, achiral essential oil profiles were largely composed of limonene (71.0%, 27.7%), *trans*-β-ocimene (3.6%, 6.9%), and pulegone (15.9%, 4.3%), respectively. Between the two species, eight chiral pairs were analyzed and, interestingly, the dominant enantiomer (calculated as ee%) of limonene and pulegone switched between the two species. Where enantiopure standards were not commercially available, MRR was used as a reliable analytical technique for chiral analysis. This study verifies the achiral profile for *A. urticifolia* and, for the first time to the authors’ knowledge, establishes the achiral profile for *M. odoratissima* and chiral profile for both species. Additionally, this study confirms the utility and practicality of using MRR for determining chiral profiles in essential oils.

## 1. Introduction

The Lamiaceae (mint) family is comprised of over seven thousand species and within the mentheae tribe are two small genera, *Agastache* and *Monardella* [1]. Both genera contain plant species that are native to the state of Utah, *Agastache urticifolia* and *Monardella odoratissima* [2,3,4,5].

*Agastache urticifolia* (Benth.) Kuntze is a perennial flowering mint that is strongly aromatic [5,6]. Two varieties of the species exist, var. *urticifolia* and var. *glaucifolia*, however, only native populations of the former exist in Utah mountain ranges [6]. Though native to the Intermountain West (USA), this species has been cultivated throughout the world [7,8,9] and even used for ethnobotanical practices in Bangladesh [10]. Scanning electron microscopy has shown that adaxial portions of the leaf contain both simple non-glandular and glandular (capitate and subsessile) trichomes from which essential oil can be extracted [11]. Previously analyzed *A. urticifolia* essential oil samples from cultivated (Russia) and native (Oregon, USA) populations have been shown to be primarily composed of the volatile compounds limonene, menthone, and pulegone [8,12], all of which are found as enantiomers in nature. To the authors’ knowledge, samples from Utah have not been previously analyzed.

*Monardella* species are endemic to western North America [13]. *Monardella odoratissima* Benth. is a small, fragrant perennial flowering mint [5,6] found in California, Colorado, Idaho, Montana, Nevada, Oregon, Utah, and Washington state [14,15,16,17]. The morphological variation of this species from distinct locations has led some researchers to classify these variations as ecotypes while others have classified them as either subspecies or varieties, so additional botanical clarification appears necessary [6,14,15,18]. To the authors’ knowledge, the essential oil composition of this species has not been previously established.

Plants in the mint family are the topic of frequent research and are of economic importance [19]. The essential oil extracted from aromatic plants in the mint family are used for flavor, fragrance, medicines, as well as other industries [20]. Due to the abundant variety of species within the mint family and the variety of volatile compounds they produce, they are widely distributed and cultivated throughout the world [20,21]. Research on the essential oil of novel plants in the mint family, as well as the development of analytical techniques to test those essential oils, is of both academic and economic interest.

The present study establishes and compares the essential oil composition, enantiomeric profile of prominent chiral compounds, and essential oil yield of *A. urticifolia* and *M. odoratissima* from Utah mountains. These data are determined utilizing conventional analytical techniques of GC/MS and GC/FID, and the novel application of chiral tagging molecular rotational resonance (MRR). MRR characterizes volatile compounds in the gas phase through their characteristic pure rotational momentum transitions [22]. As MRR is an extremely high-resolution spectroscopic technique and is highly sensitive to small changes in a molecule’s three-dimensional mass distribution, distinct compounds, including isomers, can be resolved in a mixture without chromatographic separation or the need for enantiopure reference standards [23,24]. To the authors’ knowledge, this is the first time *A. urticifolia* essential oil of Utahn origin has been researched as well as the first time the composition of *M. odoratissima* essential oil and enantiomeric profile of either species from any location have been researched. Results provide a foundation for future pharmacological research and taxonomy-chemotaxonomic clarification.

## 2. Results

The essential oils of *Agastache urticifolia* and *Monardella odoratissima* share similar compounds in their achiral composition, with both essential oils being largely composed of limonene (71.0%, 27.7%), *trans*-β-ocimene (3.6%, 6.9%), and pulegone (15.9%, 4.3%), respectively. However, the essential oil composition of *M. odoratissima* is more complex, containing 57% more detectable and identifiable volatile compounds as well as being composed of additional prominent compounds, including sabinene (27.1%), β-pinene (2.1%), myrcene (4.3%), 1,8-cineole (12.1%), and citronellol (2.3%) (Table 1).

Between the two species, eight chiral pairs were analyzed: α-pinene, sabinene, β-pinene, limonene, menthone, pulegone, α-terpineol, and citronellol (Table 2). Enantiopure reference standards were commercially available for all prominent (defined as averages ≥ 1%) chiral monoterpenoids found in these essential oils, except for sabinene. Chiral tagging molecular rotational resonance (MRR) was used to determine ee% of sabinene in *M. odoratissima* essential oil. To illustrate the similarity between values from conventional techniques (GC/MS and GC/FID) and the novel application of chiral tagging MRR for determining chiral profiles, ee% was determined for limonene in both essential oils using both techniques (Table 3). Values (ee%) compared between each technique agree within 4% or less.

Essential oil yield is detailed in Table 4. The average essential oil yield for *A. urticifolia* and *M. odoratissima* are 0.14% and 0.16%, respectively.

## 3. Discussion

The essential oils of *Agastache urticifolia* and *Monardella odoratissima* share similar properties in their achiral composition, with both essential oils being largely composed of limonene (71.0%, 27.7%), *trans*-β-ocimene (3.6%, 6.9%), and pulegone (15.9%, 4.3%), respectively. Additionally, both essential oils share 26 compounds, either prominent or non-prominent, in common with their achiral profiles. While the achiral composition of the two mint species is similar, there are stark differences in the composition. The essential oil composition of *M. odoratissima* is more complex, containing 57% more detectable and identifiable volatile compounds as well as being composed of additional prominent compounds, including sabinene (27.1%), β-pinene (2.1%), myrcene (4.3%), 1,8-cineole (12.1%), and citronellol (2.3%). Pulegone, which is prominent in both plant species, is not a common component of many essential oils. However, it is the prominent compound of *Mentha pulegium* (pennyroyal mint) [26,27].

Between the two species, eight chiral pairs were analyzed: α-pinene, sabinene, β-pinene, limonene, menthone, pulegone, α-terpineol, and citronellol. Interestingly, the dominant enantiomer of limonene and pulegone differed between the two species. Limonene values were predominantly composed of the (R)-(+) enantiomer for *A. urticifolia* (96.7 ee%) and the (S)-(-) enantiomer for *M. odoratissima* (61.6 ee%). This pattern was flipped for pulegone. Pulegone values were predominantly composed of the (S)-(-) enantiomer for *A. urticifolia* (> 99.0 ee%) and (R)-(+) enantiomer for *M. odoratissima* (> 99.0 ee%). The ee% values for *M. odoratissima* are similar to previous findings of *Mentha piperita* (peppermint), with (S)-(-)-limonene and (R)-(+)-pulegone enantiomers being prominent as well [28].

Enantiopure reference standards were commercially available for all prominent chiral monoterpenoids found in these essential oils, except for sabinene. Given the distinct capabilities of molecular rotational resonance (MRR) to resolve enantiomers through chiral tagging and quantify specific compounds, including isomers, without chromatographic separation or the need for enantiopure reference standards, this technique was used to determine ee% of sabinene in *M. odoratissima* essential oil. To illustrate the similarity of values between the conventional techniques of GC/MS and GC/FID [28,29,30] and the novel application of MRR for determining chiral profiles, ee% was determined for limonene in both essential oils using both techniques. Values (ee%) compared between each technique agree within 4% or less. The standard deviation calculated on repeat injections of the MRR measurements was higher than that of the GC measurements (Table 2, Table 3). In MRR, the precision of the measurement is determined by the integration time of the method. The methods in this study were approximately 18 min in length (including time for cleaning between samples). A longer method could be developed in the future to improve the measurement precision as needed.

The essential oils of *A. urticifolia* and *M. odoratissima* display characteristics common to the more well-known essential oils of peppermint and pennyroyal mint, in terms of both the achiral and chiral aromatic profiles. The detailed profiles provide a foundation for future pharmacological research and taxonomy-chemotaxonomic clarification; however, additional research is needed to investigate both fields.

## 4. Materials and Methods

**Figure 1:**
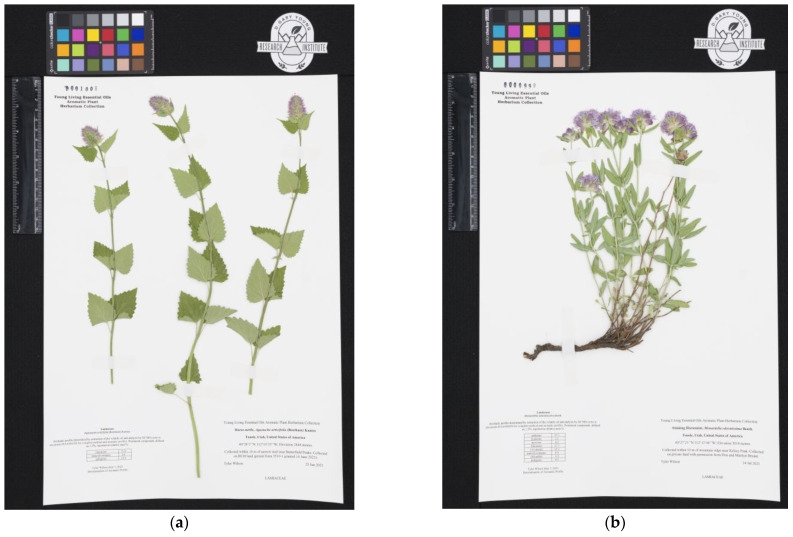
Images of voucher samples for *Agastache urticifolia* (**a**) and *Monardella odoratissima* (**b**) submitted to the Young Living Aromatic Herbarium (YLAH).

Plant material was collected during the second and third week of July 2021 near Butterfield Peaks (*Agastache urticifolia*) and Kelsey Peak (*Monardella odoratissima*) in the Oquirrh Mountain range of Utah, USA. Collection sites were on both public (*A. urticifolia*) and private land (*M. odoratissima*), with written consent for collection from proper entities. The above ground portions were sustainably collected from flowering plants at the following locations: *A. urticifolia* (40°28′2″ N 112°10′23″ W, 2844 m elevation) and *M. odoratissima* (40°27′2″ N 112°12′46″ W, 3015 m elevation). Representative voucher samples are held in the Young Living Aromatic Herbarium (YLAH): *Agastache urticifolia* (Benth.) Kuntze, Wilson 2021-01 (YLAH) and *Monardella odoratissima* Benth., Wilson 2021-01 (YLAH) (Figure 1).

Plant material was prepared for laboratory-scale distillation as follows: plant material from each species was collected, bagged, and stored at -20 ± 2 °C until steam distilled. Steam distillation was performed in triplicate, resulting in three distillations per plant species and six distillations over the course of this research project.

Laboratory-scale distillation was as follows (using a custom distillation unit): 1.5 L of water added to 2 L steam generator that fed to a 2 L distillation chamber, plant material accurately weighed and added to the distillation chamber, distillation for 1.5 h from pass-over by indirect steam, essential oil separated by a cooled condenser and Florentine flask. Each essential oil sample was filtered and stored at room temperature in a sealed amber glass bottle until analysis.

The percent yield was calculated as the ratio of the mass of processed plant material immediately before distillation to the mass of essential oil produced, multiplied by 100.

Essential oils were analyzed, and volatile compounds identified, by GC/MS using an Agilent 7890B GC/5977B MSD (Agilent Technologies, Santa Clara, CA, USA) and Agilent J&W DB-5, 60 m × 0.25 mm, 0.25 μm film thickness, fused silica capillary column. Operating conditions: 0.1 μL of sample (20% soln. for essential oils in ethanol), 150:1 split ratio, initial oven temperature of 40 °C with an initial hold time of 5 min, oven ramp rate of 4.5 °C per minute to 310 °C with a hold time of 5 min, helium carrier gas. The electron ionization energy was 70 eV, scan range 35–650 amu, scan rate 2.4 scans per second, source temperature 230 °C, and quadrupole temperature 150 °C. Volatile compounds were identified using the Adams volatile oil library [25] using Chemstation library search in conjunction with retention indices. Note that limonene/1,8-cineole elute as unresolved peaks. Their ratios were determined by the ratio of masses 41, 68, 93 (limonene), 43, 71, 81 (1,8-cineole). Volatile compounds were quantified and are reported as a relative area percent by GC/FID using an Agilent 7890B and Agilent J&W DB-5, 60 m × 0.25 mm, 0.25 μm film thickness, fused silica capillary column. Operating conditions: 0.1 μL of sample (20% soln. for essential oils in ethanol, 1% for reference compounds in ethanol, 0.1% soln. for C7–C30 alkanes in hexane), 25:1 split injection, initial oven temperature at 40 °C with an initial hold time of 2 min, oven ramp rate of 3.0 °C per minute to 250 °C with a hold time of 3 min, helium carrier gas. Essential oil samples were analyzed in triplicate by GC/FID to ensure repeatability (standard deviation < 1 for all compounds). Compounds were assigned using retention indices coupled with the retention time data of reference compounds (MilliporeSigma, Sigma-Aldrich, St. Louis, MO, USA).

Enantioselective analysis was performed on chiral monoterpenoids that had an average area % (achiral profile) ≥ 1 for each species. Essential oils were analyzed, and chiral pairs identified, by GC/MS using an Agilent 7890B GC/5977B MSD (Agilent Technologies, Santa Clara, CA, USA) and Restek Rt-β, 30 m × 0.32 mm, 0.25 μm film thickness, fused silica capillary column. Operating conditions: 0.2 μL of sample (0.5% soln. for essential oils in ethanol), 25:1 split ratio, initial oven temperature of 40 °C with an initial hold time of 20 min, oven ramp rate of 2.0 °C per minute to 140 °C with a hold time of 35 min, second oven ramp rate of 30.0 °C per minute to 230 °C with a hold time of 2 min, helium carrier gas. The electron ionization energy was 70 eV, scan range 35–650 amu, scan rate 2.4 scans per second, source temperature 230 °C, and quadrupole temperature 150 °C. Volatile compounds were identified using the Adams volatile oil library [25] using Chemstation library search. Chiral pairs were quantified and are reported as enantiomeric excess (ee %) (Equation (1)) by GC/FID using an Agilent 7890B and Restek Rt-β, 30 m × 0.32 mm, 0.25 μm film thickness, fused silica capillary column. Operating conditions: 0.2 μL of sample (0.5% or 2% soln. for essential oils in ethanol, 0.1% for enantiopure reference compounds in ethanol), 10:1 split injection, initial oven temperature at 40 °C with an initial hold time of 20 min, oven ramp rate of 2.0 °C per minute to 140 °C with a hold time of 35 min, second oven ramp rate of 30.0 °C per minute to 230 °C with a hold time of 2 min, helium carrier gas. Essential oil samples were analyzed in triplicate by GC/FID to ensure repeatability (standard deviation < 0.5 when calculating ee % for each chiral pair). Enantiopure reference standards were used for α-pinene, β-pinene, limonene, pulegone, α-terpineol (MilliporeSigma, Sigma-Aldrich, St. Louis, MO, USA) and for menthone, citronellol (Honeywell, Fluka, Charlotte, NC, USA).

(1) $$ee\;\left(\%\right)=\frac{area\;\%\;of\;predominant\;enantiomer-area\;\%\;of\;minor\;enantiomer}{area\;\%\;of\;predominant\;enantiomer+area\;\%\;of\;minor\;enantiomer}\times 100$$

Assignment of (R)-(+)-sabinene and (S)-(-)-sabinene was determined by the isoMRR spectrometer (BrightSpec, Inc., Charlottesville, VA, USA). Duplicate determination of ee% was calculated for limonene to demonstrate technique reliability. For molecular rotational resonance (MRR), high-purity neon carrier gas, seeded with the volatile compound(s) under study, was injected into a high vacuum chamber through a pulsed supersonic expansion nozzle to create a rotationally cold sample for analysis. For resolution of enantiomers, the chiral tagging technique was used, where a small chiral molecule of known enantiomeric purity was added into the carrier gas stream. In the pulsed expansion, non-covalent diastereomeric complexes were formed between the analyte and tag, which were used to distinguish the enantiomers of the analyte by MRR [31,32]. The structures of the non-covalent complexes formed in the experiment are determined by comparison of the experimentally determined rotational constants for each of the diastereomeric complexes to those calculated from quantum chemical calculations using dispersion-corrected density functional theory. After normalization of the instrument response to the two complexes using a racemic chiral tag sample, both the absolute configuration (AC) and ee% of a volatile, chiral compound can be determined by measuring the relative signals of the two complexes. In this study, the targeted isoMRR spectrometer [24,31] was used to perform the chiral tagging measurements, with a measurement cycle time of approximately 18 min for each essential oil sample. Analytes were characterized in a procedure very similar to in the previous studies [31,32]. For each measurement, 5 μL of undiluted oil was injected, and heated to 30 °C to generate vapor of the intended analytes. The tag used was (R)-propylene oxide (PO) for the limonene ee% determination, and (R)-1,1,1-trifluoropropan-2-ol (TFIP) for the sabinene ee% determination.

## 5. Conclusions

To the authors’ knowledge, this is the first time *Agastache urticifolia* essential oil of Utahn origin has been researched as well as the first time the composition of *Monardella odoratissima* essential oil and enantiomeric profiles of either species from any location have been researched.

The essential oils of *A. urticifolia* and *M. odoratissima* share similar compounds in their achiral composition, with both essential oils being largely composed of limonene, *trans*-β-ocimene, and pulegone. Pulegone, which is a prominent compound in both plant species, is not a common component of many essential oils. However, it is the prominent compound of *Mentha pulegium* (pennyroyal mint), another plant species in the Lamiaceae (mint) family.

Between the two species, eight chiral pairs were analyzed. These data were determined utilizing conventional analytical techniques of gas chromatography (GC), and, where commercial enantiopure reference standards were not available, the novel application of chiral tagging molecular rotational resonance (MRR). Upon investigating analytical technique repeatability, the standard deviation calculated on repeat injections of the MRR measurements was higher than that of the GC measurements. In MRR, the precision of the measurement is determined by the integration time of the method. The methods in this study, including time for cleaning between samples, were approximately 18 min in length. This is in stark contrast to the chiral GC method, which run time is 110 min, not including the rinse/clean between sample injections. A longer MRR method could be developed in the future to improve the measurement precision as needed. Notwithstanding, this study confirms the utility and practicality of using MRR for determining chiral profiles in essential oils. In future studies on these two mint species, additional chiral pairs (monoterpenoids < 1%; sesquiterpenoids) could also be researched to determine a complete enantiomeric profile.

The essential oils of *A. urticifolia* and *M. odoratissima* display characteristics common to two well-known essential oils in the mint family, peppermint and pennyroyal mint, in terms of both the achiral and chiral aromatic profiles. Additional research could lead to the commercial cultivation and application of *A. urticifolia* and *M. odoratissima* essential oils. Research on the essential oils of these novel plants in the mint family, as well as the development of analytical techniques to test the essential oils, is of both academic and economic interest. The essential oil extracted from these aromatic plants could potentially be used for flavor, fragrance, medicines, as well as other industries. The detailed profiles established herein provide a foundation for future use and applications of these plants, as well as taxonomy-chemotaxonomic clarification. However, additional research is needed to investigate these fields.

## Figures and Tables

**Table 1:** *Agastache urticifolia* and *Monardella odoratissima* essential oil profiles (average values, *n* = 3 per species) determined by GC/FID. The Kovat’s Index (KI), volatile compound name, and compound average area % are provided. Each essential oil sample was analyzed in triplicate to ensure repeatability (standard deviation < 1 for all compounds). Values less than 0.1% are denoted as trace (t) and those not detected in that sample as not detectable (nd). The KI values, which are from literature, were previously calculated and obtained using a linear calculation on DB-5 column [25].

KI	Compound Name	*Agastache urticifolia*	*Monardella odoratissima*
924	α-thujene	nd	0.2
932	α-pinene	0.1	1.7
946	camphene	nd	0.3
*964	2-methyl butyl propionate	nd	0.1
969	sabinene	0.4	27.1
974	β-pinene	t	2.1
979	3-octanone	nd	0.1
988	myrcene	1.9	4.3
988	3-octanol	nd	t
*1001	2-methyl butyl isobutyrate	nd	t
1002	α-phellandrene	t	nd
1014	α-terpinene	nd	0.2
1020	p-cymene	t	0.2
1024	limonene	71.0	27.7
1026	1,8-cineole	0.1	12.1
1032	*cis*-β-ocimene	0.3	1.1
1044	*trans*-β-ocimene	3.6	6.9
1054	γ-terpinene	t	0.4
1065	*cis*-sabinene hydrate	nd	0.2
1086	terpinolene	nd	0.3
1089	p-cymenene	t	nd
1095	linalool	nd	0.4
1100	2-methyl butyl-2-methyl butyrate	nd	0.1
1103	2-methyl butyl isovalerate	nd	t
1106	*cis*-rose oxide	nd	t
1119	*trans*-p-menth-2,8-dien-1-ol	t	nd
1133	*cis*-p-menth-2,8-dien-1-ol	0.1	nd
1136	*trans*-p-menth-2-en-1-ol	nd	t
1137	*trans*-limonene oxide	t	nd
1137	*trans*-sabinol	nd	0.1
1148	citronellal	0.1	0.3
1148	menthone	1.8	t
1158	isomenthone	0.6	0.9
1165	borneol	nd	0.4
*1173	isopulegone	0.2	t
1174	terpinen-4-ol	nd	0.3
1179	isomenthol	nd	0.1
1186	α-terpineol	nd	1.0
1195	methyl chavicol	t	t
1223	citronellol	0.1	2.3
1233	pulegone	15.9	4.3
1249	geraniol	nd	0.1
1249	piperitone	nd	t
1264	geranial	nd	0.1
1271	citronellyl formate	nd	0.3
1284	bornyl acetate	t	t
1298	geranyl formate	nd	0.1
1340	piperitenone	0.1	t
1350	citronellyl acetate	t	t
1374	α-copaene	nd	t
1379	geranyl acetate	0.2	nd
1387	β-bourbonene	0.1	0.3
1389	β-elemene	0.1	0.2
1392	*cis*-jasmone	nd	0.1
1417	(*E*)-caryophyllene	0.9	0.1
1419	β-ylangene	nd	0.1
1430	β-copaene	0.1	0.1
1440	*cis*-β-farnesene	t	0.2
1452	α-humulene	0.1	nd
1480	germacrene D	1.2	1.3
1500	bicyclogermacrene	nd	0.4
1505	(*E,E*)-α-farnesene	0.3	nd
1513	γ-cadinene	nd	t
1514	cubebol	t	nd
total		99.4	98.7

* KI not previously calculated [25]. Manual calculation performed using alkane standards.

**Table 2:** Enantiomeric excess of chiral monoterpenoids that had an average area % (achiral profile) ≥ 1% for *Agastache urticifolia* and *Monardella odoratissima*. Chiral ratios (calculated as ee%) determined by GC/FID for all compounds except for sabinene, which was determined by molecular rotational resonance (MRR) (average values, *n* = 3 per species). Each essential oil sample was analyzed in triplicate to ensure repeatability (standard deviation for analysis by GC/FID < 0.5 for all compounds; standard deviation for analysis by MRR 3.6 for sabinene).

Enantiomer	*Agastache urticifolia* ee%	*Monardella odoratissima* ee%
(-)-α-pinene	-	41.1
(+)-α-pinene	-	-
(R)-(+)-sabinene	-	18.0
(S)-(-)-sabinene	-	-
(+)-β-pinene	-	-
(-)-β-pinene	-	32.1
(S)-(-)-limonene	-	61.6
(R)-(+)-limonene	96.7	-
(-)-menthone	-	-
(+)-menthone	>99.0	-
(R)-(+)-pulegone	-	>99.0
(S)-(-)-pulegone	>99.0	-
(-)-α-terpineol	-	-
(+)-α-terpineol	-	49.9
(+)-citronellol	-	-
(-)-citronellol	-	>99.0

**Table 3:** Enantiomeric excess (ee%) of limonene in essential oils of *Agastache urticifolia* and *Monardella odoratissima* determined by GC/FID and molecular rotational resonance (MRR) (average values, *n* = 3 per species). Each essential oil sample was analyzed in triplicate to ensure repeatability (standard deviation for analysis by GC/FID < 0.5 for repeat injections; standard deviation for analysis by MRR 2.8 for repeat injections). Values (ee%) between techniques agree within 4% or less.

Method	Enantiomer	*Agastache urticifolia* ee%	*Monardella odoratissima* ee%
GC/FID	(S)-(-)-limonene	-	61.6
	(R)-(+)-limonene	96.7	-
MRR	(S)-(-)-limonene	-	65.6
	(R)-(+)-limonene	>95.0	-

**Table 4:** Yield data, including weight of plant material distilled (g), essential oil yield (g), and calculated essential oil yield (%). Average calculated yields for *Agastache urticifolia* and *Monardella odoratissima* are 0.14% and 0.16%, respectively. The relative standard deviation (RSD) is provided for essential oil yield for both species.

Plant Name	Plant Sample	Plant Material Weight (g)	Essential Oil Yield (g)	Essential Oil Yield (%)
*Agastache* *urticifolia*	1	387.23	0.48	0.12
	2	390.57	0.53	0.14
	3	374.39	0.62	0.17
	Average	384.06	0.54	0.14
	Standard Deviation			0.018
*Monardella* *odoratissima*	1	403.33	0.66	0.16
	2	399.44	0.63	0.16
	3	397.41	0.61	0.15
	Average	400.06	0.63	0.16
	Standard Deviation			0.004

## Data Availability

The data presented in this study are available upon request from the corresponding author.

## Sample Availability

Samples of the compounds are not available from the authors.
